# First regional evaluation of nuclear genetic diversity and population structure in northeastern coyotes ( *Canis latrans*)

**DOI:** 10.12688/f1000research.3567.1

**Published:** 2014-03-03

**Authors:** Javier Monzón

**Affiliations:** 1 Departments of Ecology & Evolution and Molecular Genetics & Microbiology, Stony Brook University, Stony Brook, NY, 11794, USA

## Abstract

Previous genetic studies of eastern coyotes ( *Canis latrans*) are based on one of two strategies: sampling many individuals using one or very few molecular markers, or sampling very few individuals using many genomic markers. Thus, a regional analysis of genetic diversity and population structure in eastern coyotes using many samples and several molecular markers is lacking. I evaluated genetic diversity and population structure in 385 northeastern coyotes using 16 common single nucleotide polymorphisms (SNPs). A region-wide analysis of population structure revealed three primary genetic populations, but these do not correspond to the same three subdivisions inferred in a previous analysis of mitochondrial DNA sequences. More focused geographic analyses of population structure indicated that ample genetic structure occurs in coyotes from an intermediate contact zone where two range expansion fronts meet. These results demonstrate that genotyping several highly heterozygous SNPs in a large, geographically dense sample is an effective way to detect cryptic population genetic structure. The importance of SNPs in studies of population and wildlife genomics is rapidly increasing; this study adds to the growing body of recent literature that demonstrates the utility of SNPs ascertained from a model organism for evolutionary inference in closely related species.

## Introduction

Historically restricted to the open deserts and plains of central and western North America, the coyote ( *Canis latrans*) ** has colonized almost the whole continent in the last 100 years, with few exceptions ( *e.g.* Long Island) ^1, 2^. The eastward range expansion was likely facilitated by widespread deforestation associated with agricultural development in the early 20 ^th^ century and by the near extirpation of eastern wolves ( *Canis lupus lycaon* or *Canis lycaon*) and red wolves ( *Canis rufus*) ^3– 5^. The range expansion into northeastern North America advanced as two primary colonization fronts: the northern route through Ontario, where coyotes hybridized with resident eastern wolves, and the southern route through Ohio, where wolves were eradicated prior to coyote expansion ^2, 5, 6^. Along with its geographic range expansion, the coyote also experienced a niche expansion by rapidly colonizing whole new biomes, including eastern temperate and boreal forests ^1^. This complex scenario of colonization provides an interesting opportunity to explore the swift formation of population genetic structure following a rapid expansion in geographic distribution and ecological niche.

Recent analyses of population structure in northeastern coyotes have described a general lack of genetic differentiation among sampling localities, except at the coarsest geographic scales. Way *et al.* ^7^ examined genetic variation and structure in a sample of coyotes from eastern Massachusetts using mitochondrial DNA (mtDNA) and eight microsatellite loci. They found no genetic structure in coyotes within Massachusetts or even within the broader region of northeastern North America. Instead, northeastern coyotes seemed to constitute one uniform population slightly differentiated from western coyotes. In another analysis of genetic variation in northeastern coyotes, Kays *et al.* ^5^ identified three coarse phylogeographic areas: Ohio, the northeast zone, and a contact zone in western Pennsylvania and New York where the colonization front from Ohio has spread into the northeastern population. Although Kays *et al.* surveyed genetic variation in a dense geographic sample of 687 coyotes, they only used one genetic marker, the hypervariable mtDNA control region. vonHoldt *et al.* ^8^ conducted a genome-wide analysis of North American *Canis* species and detected population structure in *C. latrans*, but only at the broadest continental scale. Although vonHoldt *et al.* genotyped tens of thousands of loci, they only sampled 14 northeastern coyotes, making detection of finer levels of population structure in the region very improbable. Thus, all the previous studies of population structure in northeastern coyotes have adopted one of two strategies: sampling many individuals using one (mtDNA) or very few molecular markers, or sampling very few individuals using thousands of genomic markers. A regional analysis of genetic diversity and population structure in northeastern coyotes using many samples and many nuclear molecular markers is currently lacking.

Genetic structure is a ubiquitous property of natural, domesticated, and human populations. Population genetic structure plays considerable roles in evolution, as both the basis and the consequence of local adaptation, the splitting of one species into two if the environments are markedly different, and the adaptability of a species as a whole across its range ( *i.e.*, transformation rather than speciation) ^9^. The detection of genetic structure largely depends on the type and number of molecular markers examined, their variability in the target population, the number of individuals sampled, and the spatial sampling scheme ^10, 11^. Single nucleotide polymorphisms (SNPs) have become a popular and inexpensive tool in the field of molecular population genetics. SNPs have properties that make them a superior alternative to other widely used genetic markers, such as microsatellites and mtDNA sequences, in evaluating genetic diversity and population structure ^12– 14^. Furthermore, the sampling scheme can greatly influence the location and composition of genetic clusters, especially in species that are continuously distributed across a landscape ^11^, as are northeastern coyotes. Thus the conclusion of no population structure from previous analyses based on limited individual or genomic sampling may be imprecise.

The objective of this study was to test the hypothesis that fine-scale population structure in northeastern coyotes exists, but remains undetected due to the small number of individuals or the low resolution of the genetic markers previously analyzed. I hypothesized that population structure would be detectable, at finer levels than in previous analyses, by using an array of 16 high-heterozygosity nuclear SNPs ascertained from the dog genome and a spatially dense sample of 385 coyotes. This is the first comprehensive regional survey of genetic diversity and population structure in northeastern coyotes that uses a dense geographic sampling scheme and several SNPs. This regional analysis reveals a cryptic population structure and a geographic pattern of nuclear genetic diversity that is discrepant with previous mtDNA- and microsatellite-based surveys. More generally, this study adds to the growing body of recent literature that demonstrates the utility of SNPs discovered in a model organism for evolutionary inference in wild relatives, as long as ascertainment bias is explicitly evaluated.

## Materials and methods

### Study area and sampling

**Figure 1.**
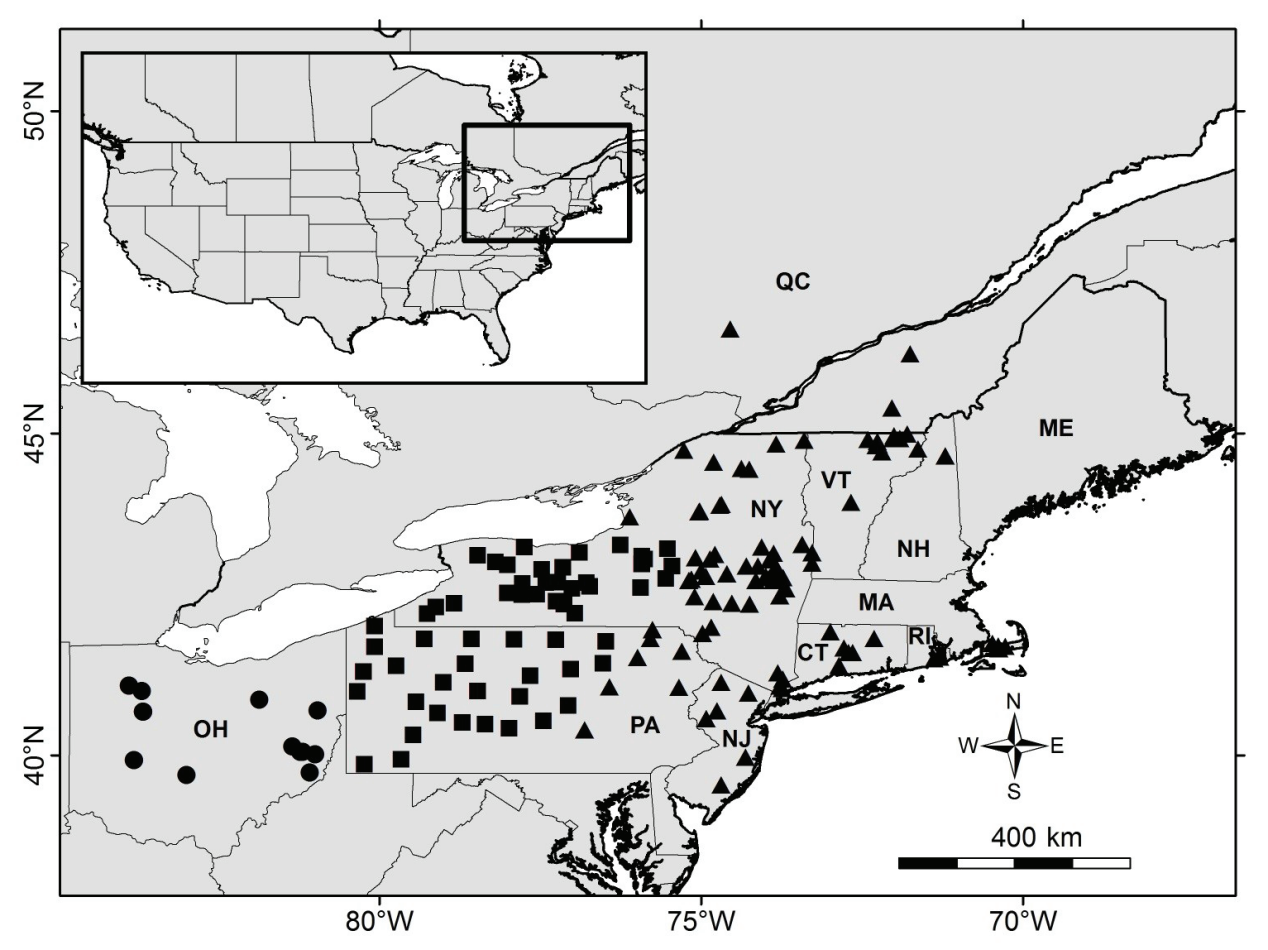
Study area and sampling localities of 385 coyotes from northeastern United States and southeastern Canada. Symbol shapes represent phylogeographic zones as in Kays *et al.* ^5^: circle, Ohio; square, contact zone; triangle, northeast zone.

The study area was located in northeastern North America. Coyotes were sampled from New York (N = 174), Pennsylvania (N = 103), Vermont (N = 34), Ohio (N = 30), New Jersey (N = 14), New Hampshire (N = 11), Connecticut (N = 8), Massachusetts (N = 5), southern Quebec (N = 4), and Rhode Island (N = 2) ( Figure 1). All samples (Total N = 385) used in this study are archived and vouchered in the New York State Museum, Albany, NY, where they were kept at -80°C. Specimens (tissues or combination of skin, skull, skeleton) were obtained primarily through donations from licensed local hunters and trappers since 1999. Six samples came from previous scat surveys in New York ^15, 16^ ( Data File). No Institutional Animal Care and Use Committee (IACUC) review was required for this study because the DNA samples came from scat or animals killed for reasons other than research.

### Marker selection and laboratory methods

I selected molecular markers based on a genomics study that used the Affymetrix Canine Mapping Array to genotype 61,435 SNP loci in hundreds of wild and domestic canids, including 14 northeastern and 3 Ohio coyotes ^8, 17^. I used the program PLINK ^18^ to compute observed and expected heterozygosity per locus in the subset of 17 northeastern and Ohio coyotes. I selected 16 unlinked SNPs, each on a different autosomal chromosome, with the highest observed heterozygosity, a measure of genetic variability in a population ( Table 1). Ascertainment schemes that select highly variable SNP loci increase power to detect population structure ^13^; accordingly, these 16 informative SNPs with high heterozygosity and high minor allele frequencies allowed me to assess genetic variation and population structure in the larger target sample. I designed primers using the Primer3 software ^19^ and tested them in silico against the dog CanFam2 genome assembly (GenBank Assembly ID: GCA_000002285.1) ^20^ using the University of California, Santa Cruz In-Silico PCR and BLAT web tools ( http://genome.ucsc.edu).

**Table 1.** Sixteen single nucleotide polymorphisms (SNPs) and their primer pairs for high-resolution melt curve genotyping assay. SNP ID chromosomal coordinates and dbSNP rs numbers correspond to the CanFam2 dog genome assembly. Measures of genetic variability correspond to the initial seventeen northeastern and Ohio coyotes ( *Canis latrans*) that formed the ascertainment panel.

SNP ID	dbSNP rs#	Forward primer	Reverse primer	PCR product size (bp)	MAF	H _O_	H _E_
chr1:92426160	rs21988674	GGGGTTTCTGAAGTGCTGAC	TGTGATAGCCACAGAAAAGCA	92	0.382	0.765	0.472
chr3:60134962	rs8795212	CACTGAGGAATGCTGGGAAG	TCAGGAAGTCTACTCCAGTGTCTG	80	0.441	0.765	0.493
chr4:33800600	rs24071674	ATCACCTCCAGAAAGCCAAG	TAAGGATCATCCCCTCGTTC	95	0.382	0.765	0.472
chr5:65740765	rs24241051	GGACCTCCATAGGACATCCA	TGTGTGGGGAGATGCAAAT	97	0.500	0.765	0.500
chr6:17110138	rs24352476	CAGTCACAATGGGGTGTCAG	AAGCGGGAGGTAGTATTACTGGT	97	0.441	0.765	0.493
chr8:69650155	rs24514604	GCTCCTGGCTATTGTATTTTCC	TTCAATTCTGCATGGTTGGT	99	0.441	0.765	0.493
chr10:45343436	rs22055760	TCTTTGAGGACATGGAACGA	TCACTCTGGAGACCAAGACG	94	0.500	0.765	0.500
chr11:66863044	rs8946304	TGGGTAATTTAATCAACGAGGAA	AAAAGCAAGAGGAGGGAACC	92	0.441	0.765	0.493
chr12:17166054	rs22184574	CAACGGCTGGATTCTGACTA	GCACACTGGTGTAGCAGAGC	118	0.441	0.765	0.493
chr16:9533917	rs22444520	TTGATAAATCAAAACCTGGGATG	GATCTGGCCCACAGCTCA	96	0.441	0.765	0.493
chr17:31508687	rs22603056	CAAAAATCAGGGATACAGACAAG	GCCAGAGAATGCCATCTTTA	100	0.471	0.824	0.498
chr19:50618604	rs22758397	TTTTTCCCTGCCTGATTTTT	TTGGAAAGAGATGTCAAGATGG	92	0.441	0.765	0.493
chr22:57259397	rs23051971	GTAGAGGACACCCTTAGATGTGG	TGTCTGGAGGGAGTTCAACA	95	0.500	0.765	0.500
chr25:44793770	rs23209441	TGACTCACCCAAGGTGATATG	CAGCTCTGATCATGCCAAAT	100	0.471	0.824	0.498
chr27:5811313	rs23365246	AATCACACACGAGCAACACC	CTGCTTGTCCTGGGATGAA	96	0.471	0.824	0.498
chr37:26421162	rs9205317	GGCTCCCAGCTAACTGTTCA	AGCTATCCAGAAGCCCAAGAG	93	0.471	0.824	0.498

**Note:** MAF: minor allele frequency; H _O_ and H _E_: observed and expected heterozygosity. Several SNPs have the same MAF, H _O_, and H _E_ due to the relatively small number of coyotes in the ascertainment panel. For example, H _O_ = 0.765 if 13 of 17 individuals were heterozygous.

I extracted total genomic DNA from muscle or hide using the DNeasy Blood and Tissue Kit (Qiagen, Valencia, USA) according to the manufacturer’s instructions, and also used DNA samples that were extracted in the New York State Museum as described in Kays *et al.* ^5^. I quantified DNA concentrations using a NanoDrop ND-1000 Spectrophotometer (Thermo Scientific, Wilmington, USA) and diluted the samples in water to attain concentrations of 5–30 ng/μl. I prepared four 96-well plates with template genomic DNA of 378 eastern coyotes, seven of which served as controls because they were already genotyped with the canine SNP microarray ^8^. Two western coyotes from Washington and two western gray wolves ( *Canis lupus*) from Yellowstone National Park served as additional controls; two wells containing only water served as negative controls.

SNP genotyping was performed at the GenoSeq Core laboratory in the University of California, Los Angeles, using a high resolution melting curve quantitative PCR assay on a LightCycler 480 thermal cycler (Roche, Indianapolis, USA). Each of the 16 SNPs was amplified and genotyped separately. DNA was amplified in a total volume of 10 μl, including 1 μl (5–30 ng) of genomic DNA, 0.2 µM of each primer, 4.2 mM MgCl _2_, and 1× Roche High Resolution Melting Master kit mix. The latter contains FastStart Taq DNA polymerase, dNTP mix, and ResoLight, a high resolution melting dye that fluoresces when DNA is double-stranded. During the melt curve analysis, the temperature increases very slowly to denature double-stranded DNA. Samples with variations in DNA sequence, even in one base pair, are distinguished by discrepancies in the shape of the melt curve, thus discriminating each of the two homozygous and the heterozygous genotypes. I processed the raw data using the Gene Scanning module of the Roche LightCycler software and followed Roche’s recommendations for evaluating data quality. The software automatically generates genotypes from the raw melt curve data. All SNPs were biallelic, so there are three possible genotypes per locus–AA, AB and BB–each forming three distinct clusters when signal intensity is plotted against temperature. I visually inspected all software-generated genotype calls and manually removed the ambiguous ones that did not conform to any of the three possible genotype clusters,

### Analyses of genetic diversity and population structure

To the 378 samples genotyped in this study, I added 7 more northeastern coyote samples genotyped by vonHoldt *et al.* ^8^ making a total sample size of 385. I used PLINK to calculate average observed and expected heterozygosity, measures of genetic diversity, and deviations from Hardy-Weinberg equilibrium in the overall sample and in each of the three phylogeographic zones inferred by Kays *et al.* ^5^ ( Figure 1). In order to assess the ascertainment bias of using dog genome SNPs to study genetic diversity and population structure in coyotes, I compared genetic variation in five subsets of canids: northeastern coyotes, western coyotes, western gray wolves, Great Lakes wolves, and dogs ( *Canis familiaris*) ^8, 17^. I calculated average observed and expected heterozygosity using all 61,435 SNPs from the microarray because the vast majority of the loci in the microarray were ascertained by dog-dog comparisons ^17^, and again using only the 16 high-heterozygosity SNPs genotyped in this study.

To assess population genetic structure, I excluded all individuals with more than five missing genotypes, resulting in 247 coyotes (16 in Ohio, 118 in contact zone, 113 in northeast zone). I used the program STRUCTURE 2.3 ^21^ to infer the most likely number of genetic populations. STRUCTURE implements a Bayesian algorithm to assign multilocus genotypes to genetic clusters by calculating the likelihood that a group of individuals constitutes a population. I tested whether finer population structure was detectable with the high-heterozygosity SNPs relative to the coarse structure detected with hypervariable mtDNA. I analyzed all 247 coyotes together to test whether more than three populations were detectable at the regional level, and then analyzed each zone separately to test whether more than one population was detectable within each zone. For all analyses, I used three replicate runs of 20,000 burn-in and 100,000 Markov chain Monte Carlo iterations, used the admixture ancestry model with correlated allele frequencies ^22^, and set the number of populations from K = 1 to K = 8. I verified that alpha and likelihood statistics reached convergence during the burn-in period for each number of populations analyzed. I used STRUCTURE HARVESTER ^23^ to evaluate the relative support for each value of K by plotting Ln P(D), the mean posterior probability of the data ^21^, and ΔK, a quantity related to the second-order rate of change of the likelihood function with respect to K ^10^. I used CLUMPP ^24^ to align and average the three replicate cluster membership coefficient matrices, and ArcMap 10 (Esri, Redlands, USA) to visualize the spatial distribution of genetic structure. I considered individuals with ancestry coefficients q > 0.8 as belonging to a specific cluster and individuals with all q < 0.8 as being admixed, consistent with recent genetic investigations of *Canis* ^25, 26^. In order to corroborate inferences from the STRUCTURE analysis with a model-free approach, I conducted a principal components analysis (PCA) using the adegenet 1.3–4 package ^27^ in R ^28^. I also computed pairwise F _ST_, the inbreeding coefficient within populations relative to the total, among the populations inferred by STRUCTURE and tested the significance of the differentiation by analysis of molecular variance (AMOVA) using 999 permutations in GenAlEx 6.5 ^29, 30^.

## Results

I interrogated 16 SNP loci in 378 coyotes for a total of 6,048 expected genotypes ( Data File). Genotyping efficacy varied by source of DNA, with fecal samples amplifying less effectively than tissue samples (Mann-Whitney test: *U* = 1771, *P* = 0.017). That is, the fecal samples had, on average, more missing or ambiguous genotypes than the tissue samples.

The overall sample of 385 eastern coyotes had lower genetic diversity than expected ( Table 2), even though observed heterozygosity generally exceeded expected heterozygosity in the ascertainment panel of 17 northeastern and Ohio coyotes ( Table 1). Ohio coyotes were the most genetically diverse in the region, but the eastward decay in genetic diversity observed with mtDNA was not replicated with nuclear SNPs. Although coyotes from the northeast zone had the lowest levels of mitochondrial genetic diversity, these same individuals had a level of nuclear genetic diversity comparable to Ohio coyotes ( Table 2). The most pronounced differences between observed and expected heterozygosity occurred in the contact zone and in the overall regional analysis ( Table 2). In the overall sample of 385 eastern coyotes, five loci were in Hardy-Weinberg equilibrium. The number of loci in Hardy-Weinberg equilibrium increased when each phylogeographic zone was analyzed separately: 13 in Ohio, nine in the contact zone, and nine in the northeast zone ( Table 2).

**Table 2.** Genetic diversity of eastern coyotes ( *Canis latrans*) measured with mtDNA sequences and 16 nuclear SNP genotypes. Most individuals genotyped at 16 nuclear SNPs represent a subset of those individuals sequenced.

Zone	N	mtDNA control region		High-heterozygosity SNPs			
		Haplotype diversity	θ (per site)	N	H _O_	H _E_	HWE
Ohio	30	0.844	0.018	30	0.465	0.435	13
Contact	207	0.721	0.014	177	0.312	0.411	9
Northeast	450	0.664	0.008	178	0.442	0.457	9
Total	687	0.708	0.013	385	0.388	0.444	5

**Note:** mtDNA data and zone designations from Kays *et al.* ^5^. N: sample size; H _O_ and H _E_: observed and expected heterozygosity; HWE: number of loci in Hardy-Weinberg equilibrium.

Dogs appeared to be the most genetically diverse when the diversity of the five different canid groups was estimated using all 61,435 SNPs from the canine microarray. The genome-wide ascertainment bias was towards dogs: the expected heterozygosity of dogs was almost twice that of western coyotes. But the ascertainment bias reversed when heterozygosity was measured using only the 16 selected SNPs: coyotes appeared to be the most genetically diverse, with northeastern coyotes having a very high expected heterozygosity, whereas dogs appeared the least genetically diverse ( Table 3).

**Table 3.** Ascertainment bias of surveying genetic diversity in different groups of canids using SNPs discovered after completion of the dog genome assembly.

Canid group	N	61,435 SNPs		16 high- heterozygosity SNPs	
		H _O_	H _E_	H _O_	H _E_
Northeastern coyotes	14	0.190	0.202	0.763	0.493
Western coyotes	45	0.147	0.182	0.387	0.399
Great Lakes wolves	19	0.187	0.217	0.278	0.290
Western gray wolves	32	0.203	0.238	0.271	0.319
Dogs	50	0.234	0.359	0.270	0.387

**Note:** Data for 61,435 SNPs from vonHoldt *et al.* ^8^. N: sample size; H _O_ and H _E_: observed and expected heterozygosity.

**Figure 2.**
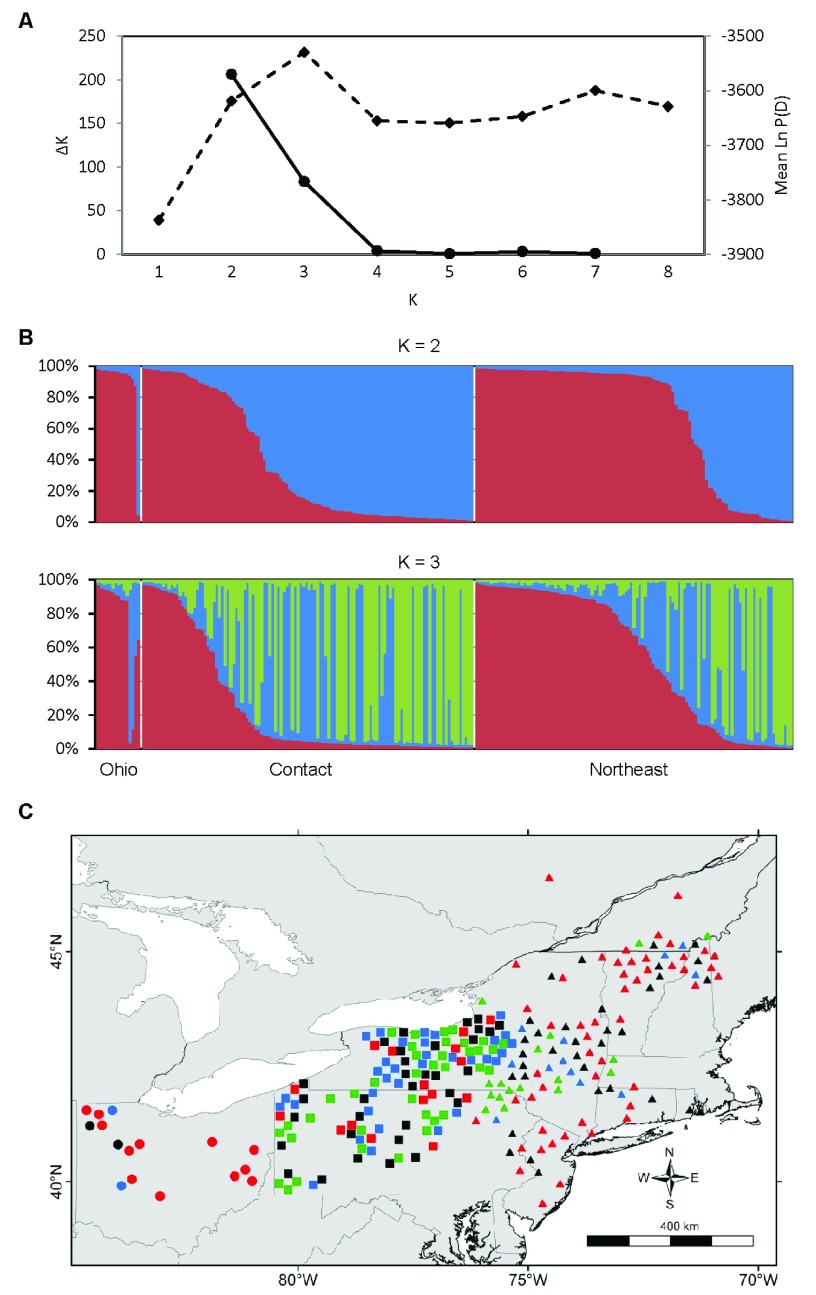
Regional population genetic structure of eastern coyotes. **A**) Estimation of the number of genetic populations using two criteria: ΔK (solid line) and Ln P(D) (dashed line). The most probable number of populations is the value of K with the maximal value of ΔK and Ln P(D). The data strongly support K = 2 and K = 3. **B**) STRUCTURE bar plots of N = 247 coyotes subdivided into K = 2 and K = 3 genetic populations; each individual is represented by a vertical bar partitioned into two or three colored segments indicating that individual’s proportional membership in each of two or three genetic clusters. **C**) Spatial distribution of K = 3 genetic populations. Symbol colors represent the genetic cluster with > 80% assignment from panel B, or black if highly admixed ( *i.e.*, no assignment > 80%). Symbol shapes represent phylogeographic zones as in Figure 1. Symbol locations are slightly jittered to display each individual and to reduce clutter.

**Figure 3.**
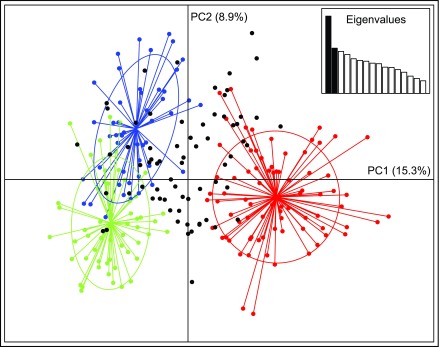
Principal components analysis of 16 autosomal SNPs genotyped in 247 eastern coyotes. Symbol colors correspond to K = 3 STRUCTURE-inferred clusters with > 80% assignment, or black if highly admixed ( *i.e.*, no assignment > 80%), as in Figure 2C. Inset shows scree plot of eigenvalues.

In the region-wide population structure analysis of 247 individuals with little missing data, the values of K with the strongest statistical support were K = 2 and K = 3 ( Figure 2A, B). Pairwise F _ST_ among the three genetic clusters varied from 0.08 to 0.10 and all were significant in the AMOVA framework ( *P* = 0.001). This indicates that there are three primary genetic subdivisions in the broad sampling area. The three groups did not correspond to the Ohio, contact, and northeast zones previously inferred by mtDNA ( Figure 2C). There is some geographic structuring, but the three groups overlap extensively in space. Although the red cluster in Figure 2B includes most of the Ohio coyotes, it is more cosmopolitan, also including many coyotes from the contact and northeast zones. The green cluster is mostly restricted to the contact zone, but extends slightly into eastern New York and Vermont. The PCA corroborated the results from STRUCTURE ( Figure 3). The first two PCA axes explained 24.2% of the total variance and clearly separated the three STRUCTURE-inferred clusters. Most admixed individuals with no clear membership in any STRUCTURE-inferred cluster also showed no clear association with any PCA cluster.

**Figure 4.**
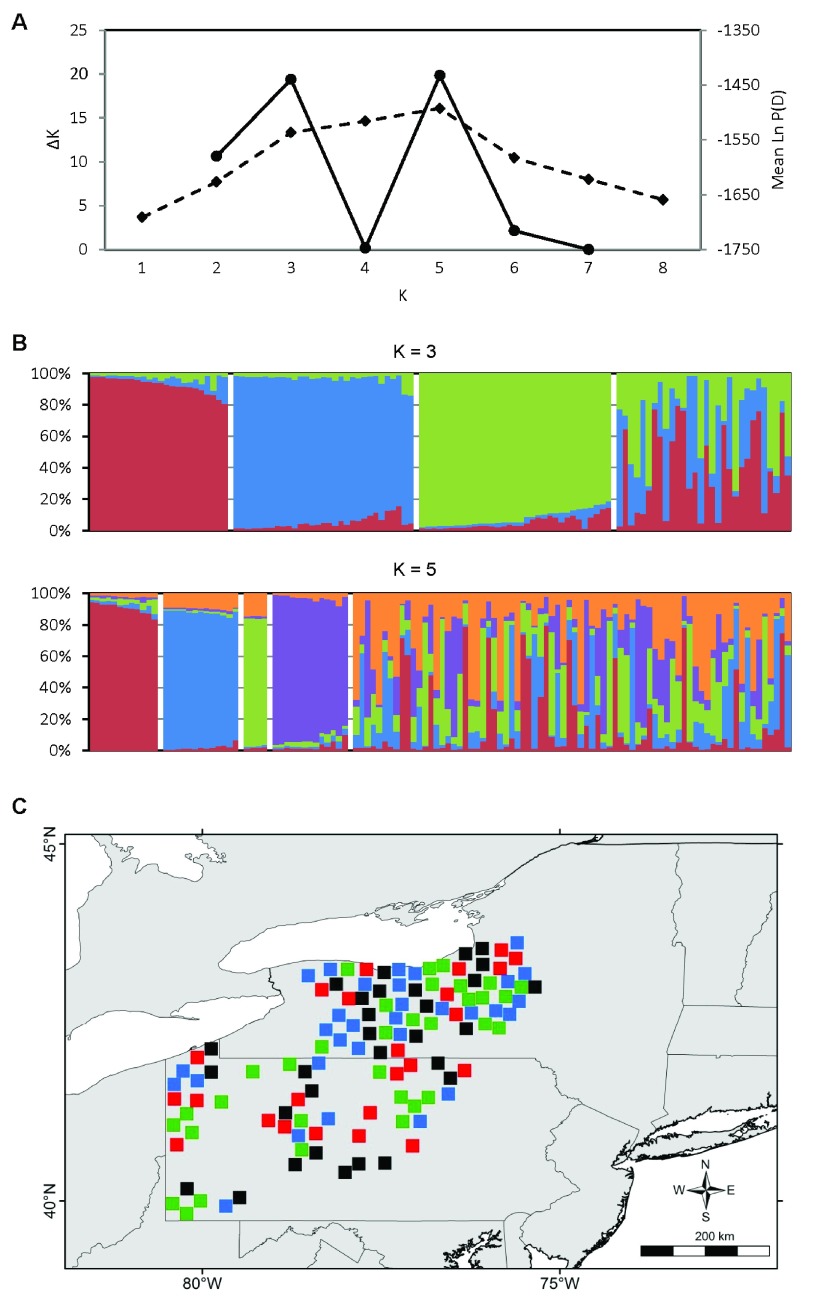
Population genetic structure of eastern coyotes in the contact zone. **A**) Estimation of the number of genetic populations using two criteria: ΔK (solid line) and Ln P(D) (dashed line). The data strongly support K = 3 and K = 5. **B**) STRUCTURE bar plots of N = 118 coyotes subdivided into K = 3 and K = 5 genetic clusters. **C**) Spatial distribution of K = 3 genetic clusters. Symbol colors represent the genetic cluster with > 80% assignment from panel **B**, or black if highly admixed ( *i.e.*, no assignment > 80%).

**Figure 5.**
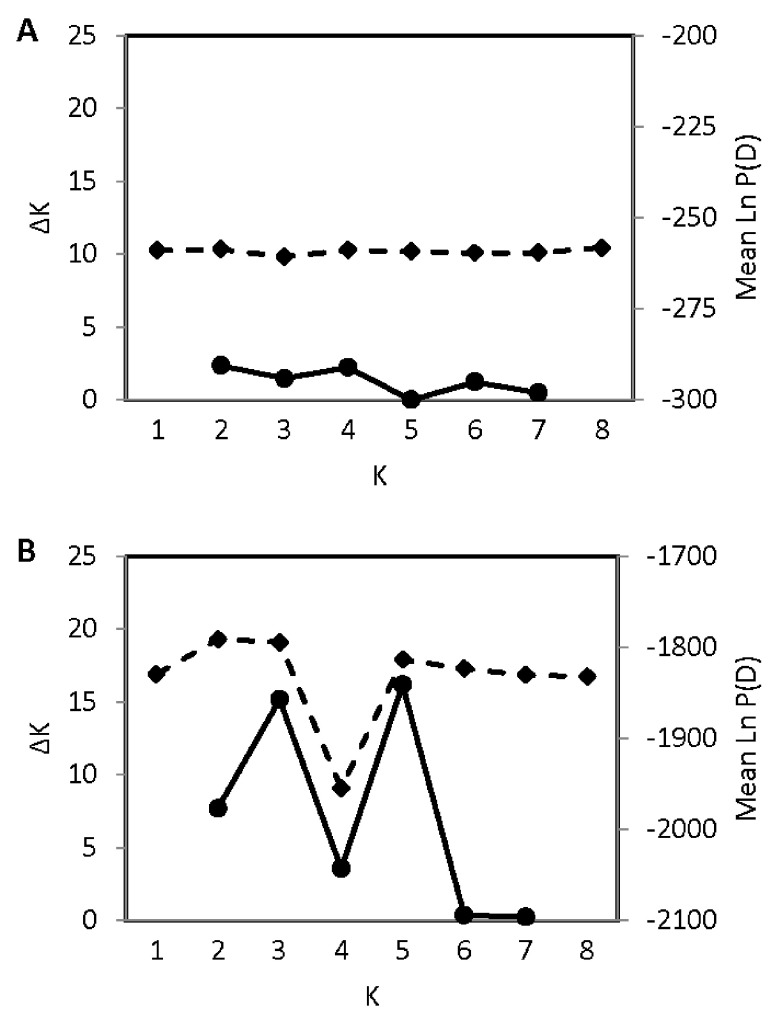
Absence of fine-scale population genetic structure in **A**) N = 16 coyotes from Ohio, and **B**) N = 113 coyotes from the northeast zone. See Figure 1 for zone designations.

Ample population genetic structure was detected in the contact zone; the values of K with the strongest statistical support were K = 3 and K = 5 ( Figure 4A). However, the K = 5 structure seems biologically unrealistic, characterized by highly admixed individuals of the various “populations” ( Figure 4B). Alternatively, the K = 3 structure identified for the contact zone was virtually identical to the regional K = 3 structure ( Figure 4C). In contrast to the contact zone, no fine-scale genetic structure was detected in Ohio or in the northeast zone. In Ohio, the value of K with the highest explanatory power was K = 1 ( Figure 5A); in the northeast zone, the value of Ln P(D) does not increase beyond K = 2 and the change in Ln P(D) between K = 1 and K = 2 is minimal, indicating weak support for genetic structure ( Figure 5B).

Data of genetic diversity in northeastern coyotes HRM_samples.csv: Specimens whose genetic profiles were analyzed
The New York State Museum specimen identifier, phylogeographic zone, state or province, coordinates in decimal degrees, year of sampling and DNA source are given for each of 385 unique eastern coyotes. Column “Structure” indicates which specimens were included in STRUCTURE analyses.
HRM_genotypes.csv: Genotypes from the Roche LightCycler 480 high-resolution melt curve assay
NYSMID: New York State Museum specimen identifier; Zone: phylogeographic zone; AA, AB, BB: genotypes for each of 16 SNPs; -: missing genotype.

## Discussion

This study documents the presence of fine-scaled population genetic structure in eastern coyotes. Specifically, the contact zone exhibits a strong signal of population structure, the same signal detected in the regional analysis. This pattern may reflect the recent merging of two colonization fronts and the highly heterogeneous landscape of New York and Pennsylvania. A separate, spatially-explicit assessment with an independent set of SNPs demonstrated that northeastern coyotes exhibit a strong signal of population structure in Central New York ^1^. Furthermore, the same investigation showed that population structure is partially explained by ecological factors, such as deer density and human land use ^1^. Indeed, eastern coyotes living in areas of high deer density are genetically more wolf-like than those living in areas of low deer density ^31^.

In contrast to the contact zone, coyotes in Ohio appear to make up a single panmictic population, as do coyotes in the northeast zone. Given the high mtDNA and nuclear diversity observed in Ohio by Kays *et al.* ^5^ and in this study, it is unlikely that the lack of genetic structure stems from a founder effect. The failure to uncover more than one genetic population in Ohio may be due instead to its landscape homogeneity or to the lack of resolution afforded by 16 individuals to detect finer levels of structure. On the other hand, the failure to uncover more than one genetic population in the northeast zone is surprising given the vast geographic area with its ecological heterogeneity and the large number of individuals sampled. Coyotes in the northeast zone are likely the descendants of a few founders and therefore do not exhibit a strong signal of population genetic structure.

The perception of population structure, even in highly vagile animals where it was least expected, has been refined by steadily improving molecular data and geographic sampling. Initially, using mtDNA restriction site polymorphisms and nuclear microsatellites, no evidence of population structure or isolation by distance was found in coyotes, even at the continental scale ^32, 33^. Various behavioral and historical explanations have been invoked to explain these early genetic patterns. But a more likely explanation is that the patterns of weak differentiation were artifacts of sparse geographic sampling or poor resolution due to the use of few molecular markers. More recent studies employing advanced analyses of spatial and genetic data have revealed strong differentiation among parapatric populations of coyotes and wolves, even in the absence of physical barriers to movement ^1, 34– 38^. These investigations used multiple loci and dense geographic sampling to uncover cryptic genetic subdivisions. Strong genetic differentiation between adjacent populations of coastal and inland wolves in British Columbia was shown with mtDNA ^39^, demonstrating that fine-scale genetic differentiation can be detected with denser sampling alone, even using a single molecular mtDNA marker. Similar cryptic subdivisions have been discovered in several highly mobile groups, such as *Lynx* ^40, 41^, ungulates ^42– 44^, cetaceans ^45, 46^, and hawks ^47^. In all these cases, genetic subdivisions appear to emerge from ecological factors and local foraging adaptations. Future studies should focus on the ecological mechanisms underlying the cryptic genetic structure in northeastern coyotes, especially because they have only inhabited the region for the last 30–80 years ^2^. Confirming ecological determinants of population structure in the absence of obvious physical dispersal barriers would provide an interesting example of rapid ecological differentiation.

There are some important similarities and discrepancies between mtDNA and autosomal SNP patterns. The data indicate that coyotes in Ohio are the most genetically diverse in the region when surveyed with nuclear SNPs, as with mtDNA ^5^. However, the gradual eastward decay in genetic diversity observed with mtDNA is not replicated with nuclear SNPs. The marked reduction of heterozygosity in the contact zone and in the overall region is very likely caused by population structure, *i.e.*, the Wahlund effect. This interpretation is supported by the increase in the number of loci in Hardy-Weinberg equilibrium at smaller geographic scales and by the congruent signal of population structure in the overall region and in the contact zone. In addition, the three primary populations detected in this study do not correspond to the three subdivisions inferred with mtDNA ^5^. Together, these results suggest that studies based solely on mtDNA should be interpreted cautiously. For example, mtDNA sequence similarity suggested that a small population of Scandinavian wolves was founded by individuals released from Swedish zoos, but nuclear polymorphic markers falsified the release hypothesis and instead supported a hypothesis of natural immigration or expansion from an unknown relict wolf pack ^48^. Discrepancies between patterns observed with mtDNA and nuclear DNA may be caused by true organismal processes, such as sex-biased dispersal ^49^. However, there is no evidence for sex-specific dispersal behaviors in eastern coyotes, consistent with their monogamous breeding system ^50, 51^. Alternatively, discrepancies may be caused by marker-specific phenomena such as effective population size, lineage sorting, mutation rate, and coalescent times ^52^, or the violation of certain assumptions of mtDNA inheritance, such as recombination, paternal leakage, and heteroplasmy ^53^. Future studies should further evaluate these sources of discrepancies.

The present study underscores three related methodological issues that are of broad interest, especially as SNPs continue to be in vogue in population and wildlife genomics. First, these data confirm that SNPs discovered in a model organism are an appropriate tool to address various questions regarding the ecology and evolution of non-model relatives. The sequencing of the dog genome ^20^ quickly enabled SNP-based investigations into wild members of the family Canidae, including coyotes, wolves, jackals, and foxes ^8, 54– 57^. Second, this study highlights the importance of evaluating the ascertainment bias of markers employed in a survey of genetic variation, especially in multi-species comparisons. Many SNP-based studies are not addressing the issue of ascertainment bias ^14^. In the present study, the genome-wide analysis of variation is dog-biased because SNPs were ascertained primarily from comparisons of boxer and poodle genomes after the completion of the dog genome project ^8^. On the other hand, the high-heterozygosity SNP analysis is coyote-biased because the 16 SNPs were chosen from an ascertainment panel of northeastern coyotes. The bias in the latter case is not problematic because the ascertainment panel from which the loci were selected is representative of the whole population of northeastern coyotes. However, the diversity measures reported in this study should not be compared to similar measures from other populations, unless the comparison corrects for ascertainment bias ^58^. Lastly, this study emphasizes the necessity of selecting SNPs very carefully to match the research question of interest. Here, a set of high-heterozygosity SNPs was interrogated in order to examine geographic patterns of genetic diversity and population structure. But other research questions may require polymorphic markers with other properties. For example, in order to better understand the complex hybrid ancestry of the northeastern coyote, Monzón *et al.* ^31^ used species-diagnostic SNPs to quantify the relative contributions of its parental populations. In addition, recent advances in the molecular genetics of phenotypic traits in dogs allow the use of SNPs linked to genes of known function to address long-standing questions about morphological, physiological, and behavioral adaptations in northeastern coyotes and other wild canids ^1, 59^.

## Data availability

*figshare*: Data of genetic diversity in northeastern coyotes. http://dx.doi.org/10.6084/m9.figshare.943483 ^60^
