# Supplementary material for: First regional evaluation of nuclear genetic diversity and population structure in northeastern coyotes ( Canis latrans)
Source: F1000Res. 2014 Mar 3;3:66. [Version 1] doi: 10.12688/f1000research.3567.1 (PMC4097358; doi:10.12688/f1000research.3567.1)
Supplement: Peer review history

# Peer review: Referee response for version 1

**DOI:** 10.5256/f1000research.3819.r3945

**Published:** 2014-05-30

**Authors:** Mark Weckel

**Affiliations:** 1 Center for Biodiversity & Conservation, American Museum of Natural History, New York, NY, USA

First, thank you for the opportunity to review Javier Monzón’s article, “ *First regional evaluation of nuclear genetic diversity and population structure in northeastern coyotes (Canis latrans*).”

I found this to be a well-written, engaging, and thoughtful manuscript with important contributions to the use of SNPs in studies of population structure and in elucidating the genetic structure of the northeastern coyote. The author adeptly targets a need for using multiple markers across multiple individuals for proper inference. The author furthermore supports the use of the canine SNP array in studies of coyotes, following the recommendation of vonHoldt *et al.* (2011), while also asserting the need for addressing ascertainment bias prior to the use of SNPs in non-model organisms.

I have no major reservations, rather a few minor comments regarding clarification of the methods along with some questions and considerations for future discussion and research.

**Methods:**

1. With such a large sample of high quality tissue available for the analysis, why were scats (n=6) included in the analysis? Were the scats important for adequate sampling across the study area?
2. Fourteen of vonHoldt *et al’s*. (2011) coyote specimens were included in the initial selection of the 16 SNP panel used in subsequent analyses. Why were only 7 of vonHoldt *et al.’s* (2011) samples included in the analysis of diversity and structure?

**Results:**

1. Using the SNP panel, the author reports the observed and expected heterozygosity for the three putative population zones identified by Kays *et al.* (2010; Table 2). It would be interesting to see the Ho and He for the population clusters identify by the current STRUCTURE analysis (i.e., K =2 & K=3).
2. What are the pairwise Fst values for two genetic clusters also supported by the STRUCTURE analysis?

**Discussion:**

1. There is ample evidence of genetic structure; however, I’m not sure that I would agree with the author’s suggestion that there is “ *some geographic structuring,*” at least based on Figure 2c. The author does a thorough job of listing the factors and processes that may be involved in explaining a lack of geographic structure, as well as the discrepancy between the current nuclear analysis of population structure and the previous mtDNA analysis (Kays *et al.* 2010).
   
  - A possible further explanation for the lack of geographic structuring could be that a SNP panel - with such high heterozygosity - may actually reflect older mutations that have had a longer time to move around the landscape via dispersal (see Morin *et al.* 2004). This might allow one to detect patterns of genetic structure without any obvious geographic patterns.
  - The author also mentions, correctly, that based on the current state of knowledge, there is no sex-bias in coyote dispersal. I wonder if the author’s data set could be used to investigate this by factoring in sex and rerunning the analyses presented in this manuscript. 

I have read this submission. I believe that I have an appropriate level of expertise to confirm that it is of an acceptable scientific standard.

# Peer review: Referee response for version 1

**DOI:** 10.5256/f1000research.3819.r3944

**Published:** 2014-05-01

**Authors:** Linda Rutledge

**Affiliations:** 1 Biology Department, Trent University, Peterborough, ON, Canada

This is a very well-written, interesting article on utilizing highly-polymorphic SNPs to identify cryptic substructure in a population of eastern coyotes. The analyses are comprehensive (including Bayesian cluster analysis, principal components analysis, and Fst calculations) and the data do seem to support fine-scale population substructure. If it weren't for the extensive analyses I would find it very difficult to believe that 16 SNPs (even highly polymorphic ones) could find such fine-scale structure because it has been estimated that it take at least 15x more SNPs than microsatellites to identify structure in populations ( Haasl & Payseur 2011, *Heredity*). Presumably the same clustering patterns would remain if the samples were analyzed at thousands of genome-wide SNPs that have not been selected for their polymorphism - i.e. presumably more, less polymorphic loci should show the same substructure.

The biological/ecological reason for the clustering remains unclear (but that is clearly beyond the scope of the paper and is really quite difficult to tease apart). The author provides plausible explanations for the structure (e.g. " *merging of the two colonization fronts*"; possible deer or human density patters), but I wonder if social structure (i.e. family/pack groups) may contribute to the genetic patterns found.  

Overall, the manuscript reads very well and provides a rigorous analysis of a novel dataset that complements the authors other work.

I have read this submission. I believe that I have an appropriate level of expertise to confirm that it is of an acceptable scientific standard.

# Peer review: Referee response for version 1

**DOI:** 10.5256/f1000research.3819.r3946

**Published:** 2014-03-18

**Authors:** Astrid Stronen

**Affiliations:** 1 Department of Biotechnology, Chemistry and Environmental Engineering, Aalborg University, Aalborg, Denmark

Thank you for the opportunity to review this manuscript. Javier Monzón presents a timely and relevant study of population structure in Northeastern coyotes, which complements earlier work in this area and highlights new and intriguing research questions. The author builds on previous genomic analyses of canids with a high-density SNP chip yet a limited sample of Northeastern coyotes, and a study of northeastern coyotes that used several hundred samples but a single genetic marker. The panel of selected SNPs is biased toward the study population of Northeastern coyotes, but the author makes this very clear and includes an evaluation of ascertainment bias across canid groups included in the initial study with the high-density SNP chip from which this panel of 16 loci was selected.

The article is well written, and the author has done a good job explaining the main limitations of the work and outlining why findings should be interpreted with caution. I have one suggestion on supplementary data that could be included for an additional perspective on genetic structure, and some minor comments on the manuscript text:   

1. The author describes the SNP results relative to the areas of Ohio, the contact zone and the northeast zone from Kays *et al.* 2010, and in Table 2 notes that “ *most individuals genotyped at 16 nuclear SNPs represent a subset of those individuals sequenced*” for mtDNA. If individual mtDNA results are available for coyotes, it would be valuable to see the extent to which the mtDNA haplotypes classified as Great Lakes wolf (or eastern wolf), coyote and gray wolf from Kays *et al.* 2010 (Fig 2) correspond with genetic structure based on SNPs.This would provide important information to help understand how similarities/discrepancies in genetic structure are distributed across space. 
             
2. The relatively low genetic diversity for coyotes in Table 3 and the results from vonHoldt *et al.* 2011 suggest a bias against coyotes, although coyotes may at least in some regions, have higher genetic diversity than wolves considering their range expansion and increase in population size. It might be worth noting that sequencing coyotes directly could provide a more in-depth view of genomic regions under selection. Although I agree with the author’s conclusion about the utility of using a closely related and well-studied species, alternate methods such as genotyping-by-sequencing could be recommended as possibilities for future research.  
3.  Introduction, 1 ^st^ sentence “ *Historically restricted to the open deserts and plains*...”. Though the coyote is widely regarded as a species of western and central North America, it is difficult to exclude the possibility that it could also have been present farther east prior to European colonization and landscape modifications. 
4. Introduction, 3 ^rd^ paragraph:
  - The sentence “ *Population genetic structure plays considerable roles in evolution... the splitting of one species into two if the environments are markedly different*”.  In addition to adaptation, it may be important also to mention genetic drift, and subsequent development of incompatibilities between isolated populations.
  - The sentence starting with “ *The detection of genetic structure largely depends on the type and number of molecular markers examined*”: Here you may want to mention explicitly the temporal resolution of markers with different mutation rates (as you have done in the Discussion).
  - “ *SNPs have become a popular and inexpensive tool*”: perhaps “ *increasingly affordable*” or similar would be better here?    
5. Discussion, 3 ^rd^ paragraph, sentence: *“...cryptic genetic structure of Northeastern coyotes, especially as they have only inhabited the region for 30-80 years*”. The author notes earlier in the text that coyotes in the northeast zone may descend from a few founders, which likely arrived via a northern route through Ontario, whereas a second colonization front occurred farther south through Ohio. Bierne *et al.* 2011 proposed that intrinsic (environment-independent) genetic incompatibilities may coincide with ecological boundaries, as these environmental transition zones often “trap” the tension zones resulting from e.g. assortative mating. Such processes might be influencing the contact zone between the southern and northern colonization fronts, and interact with the selective pressures associated with forested, agricultural and urban habitat, and deer density, noted in Monzón 2012 (PhD Dissertation).    

I have read this submission. I believe that I have an appropriate level of expertise to confirm that it is of an acceptable scientific standard.
